# Supplementary material for: Proteomic method to extract, concentrate, digest and enrich peptides from fossils with coloured (humic) substances for mass spectrometry analyses
Source: R Soc Open Sci. 2019 Aug 21;6(8):181433. doi: 10.1098/rsos.181433 (PMC6731700; doi:10.1098/rsos.181433)
Supplement: Supplementary Images [file rsos181433supp1.pdf]

Supplementary Information: A proteomic method to extract, concentrate, digest, and enrich peptides from fossils with colored (humic) substances for mass spectrometry analyses

Elena R. Schroeter,[1]* Kevin Blackburn,[2] Michael B. Goshe,[2] Mary H. Schweitzer[1]

1. Department of Biological Sciences, North Carolina State University, Raleigh, NC 27513, easchroe@ncsu.edu
2. Department of Molecular and Structural Biochemistry, North Carolina State University, Raleigh, NC 27513

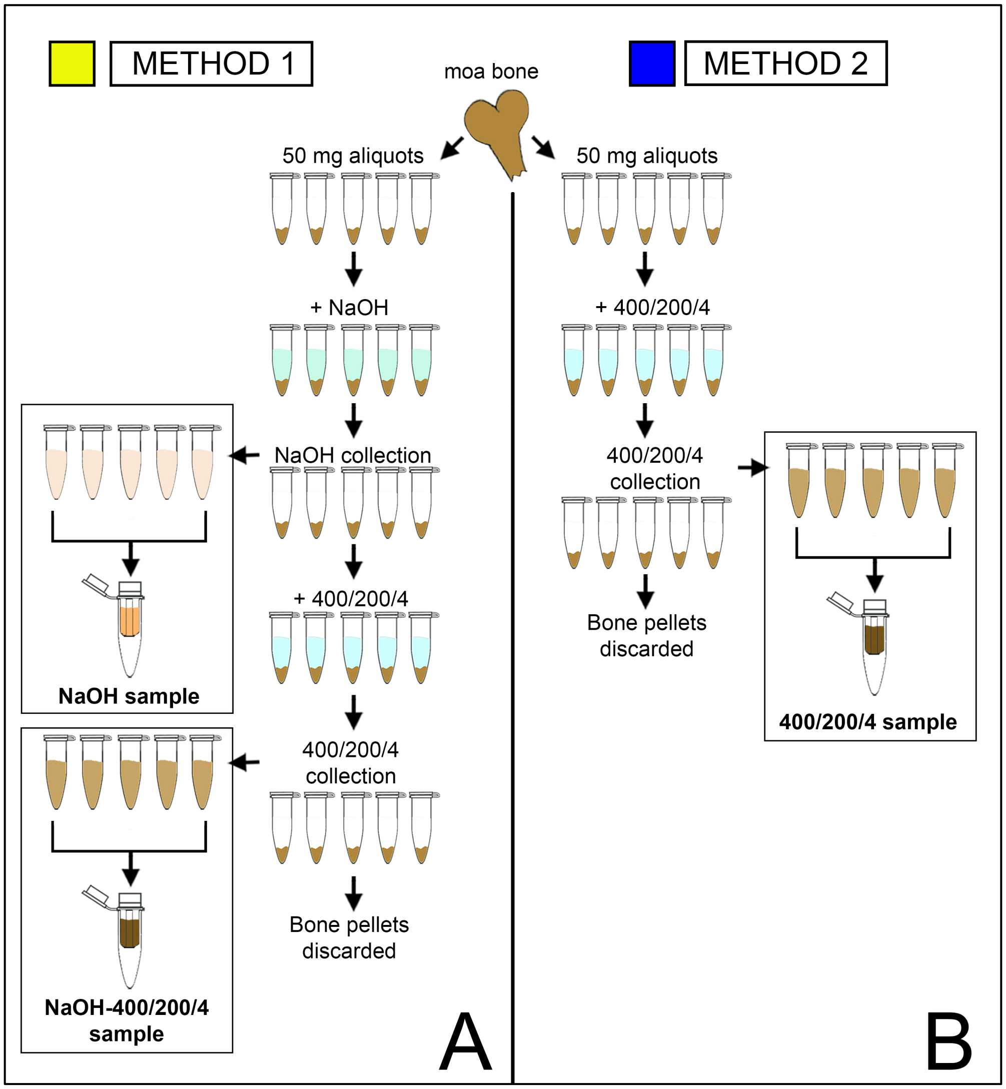

Figure S1. Diagram of proteomic samples generated by this study. (A) Method 1: 5 aliquots of 50 mg ground moa bone powder were incubated in 0.1 M NaOH. The NaOH supernatant was subsequently collected and concentrated into a single sample, "NaOH." Bone pellets were then incubated in 400/200/4 solution, which was collected and concentrated into a second sample, "NaOH-400/200/4." This label denotes that it was generated from bone that received 400/200/4 *after* pretreatment with NaOH. (B) Method 2: 5 aliquots of 50 mg ground moa bone powder were incubated directly in 400/200/4. The 400/200/4 supernatant was subsequently collected and concentrated into a solitary sample, "400/200/4."

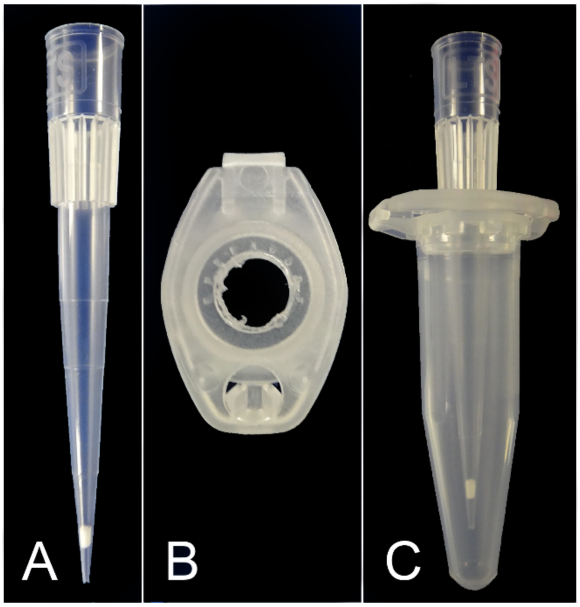

Figure S2. Stage tip assembly. (A) Self-made stage tip, with 2 discs of C18 membrane inserted in a 200 µl pipette tip. (B) Centrifugation tube that has been perforated through the lid with a heated glass Pasteur pipette. For Protein LoBind Eppendorf 1.5 mL tubes, the central ring printed on the lid can be used as a guide. (C) Stage tip inserted in perforated tube. This assembly, adapted from Yu et al.[21], allows multiple samples to be stage tipped simultaneously and in a uniform manner.

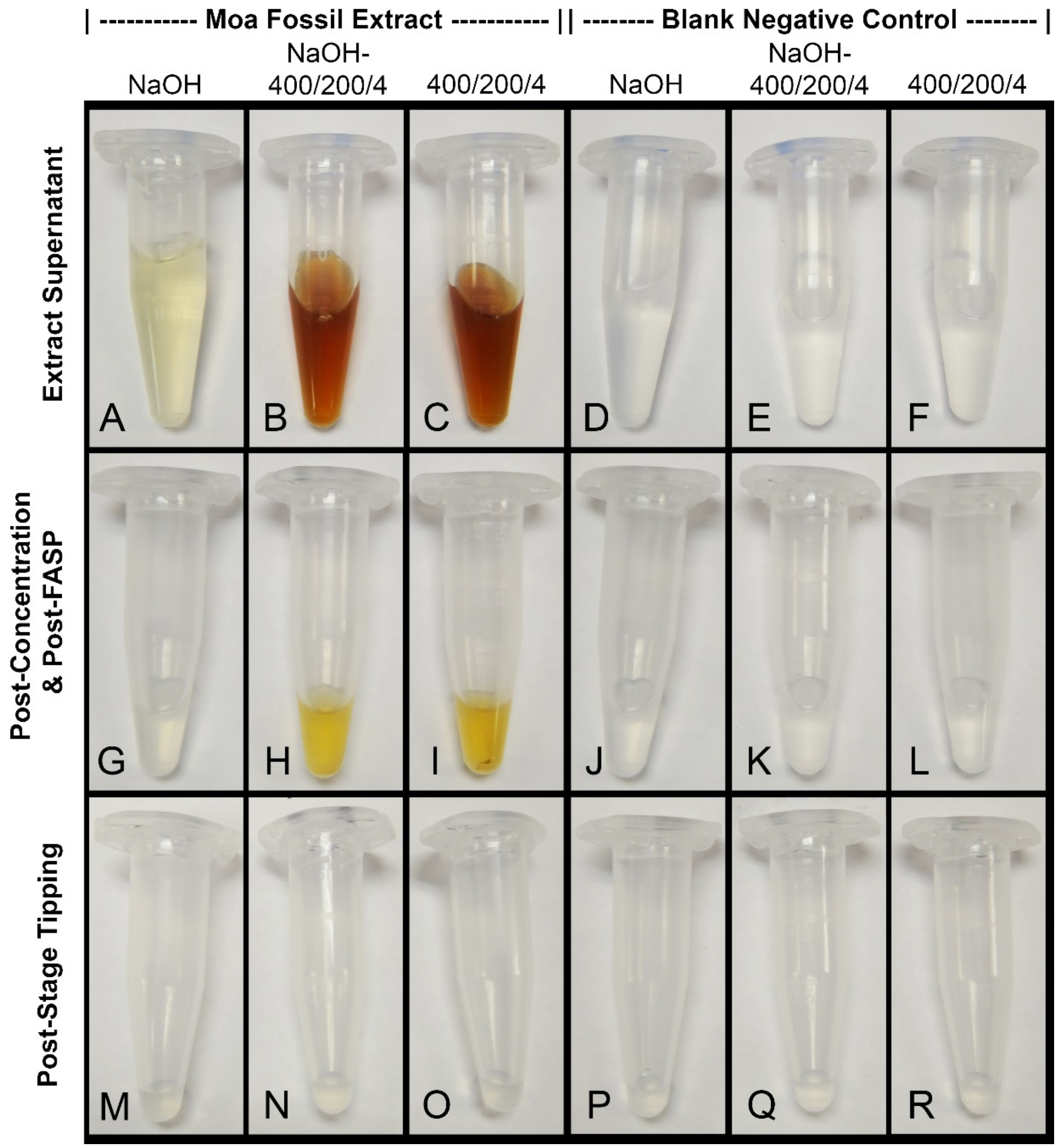

Figure S3. Progression of extracts through preparation for MS. (Row 1: A-F) Supernatant (NaOH or 400/200/4) immediately upon collection. Each tube represents one of five that were subsequently concentrated together. (Row 2: G-L) Supernatants after concentration and FASP using 3 kDa MWCO filters. Despite combining five tubes with high humic content, passing samples through the filter after digestion has removed a large portion of the high-weight humics from the smaller peptides. (Row 3: M-R) Supernatants after stage tipping. Upon elution from the stage tips, samples are now as colorless as blank controls, suggesting that most inferring humic substances have been removed. (A,G,M) NaOH extract from moa bone; (B,H,N) 400/200/4 extract from moa bone previously treated with NaOH; (C,I,O) 400/200/4 extract from untreated moa bone; (D,J,P) NaOH extract from empty tubes (blank negative controls); (E,K,Q) 400/200/4 extract from empty tubes previously filled with NaOH; (F,L,R) 400/200/4 extract from empty tubes. Note: the tubes pictured under "Moa Fossil Extract" are the same ones appearing in Figure 2, but are shown here for ease of comparison to those pictured under "Blank Negative Control."
